# Specific MHC class I supertype associated with parasite infection and color morph in a wild lizard population

**DOI:** 10.1002/ece3.4479

**Published:** 2018-09-17

**Authors:** Jessica D. Hacking, Devi Stuart‐Fox, Stephanie S. Godfrey, Michael G. Gardner

**Affiliations:** ^1^ College of Science and Engineering Flinders University Bedford Park South Australia Australia; ^2^ School of BioSciences University of Melbourne Parkville Victoria Australia; ^3^ Department of Zoology University of Otago Dunedin New Zealand; ^4^ Evolutionary Biology Unit South Australian Museum Adelaide South Australia Australia

**Keywords:** Agamidae, *Ctenophorus decresii*, major histocompatibility complex, MHC‐associated mating, parasite‐mediated selection

## Abstract

The major histocompatibility complex (MHC) is a large gene family that plays a central role in the immune system of all jawed vertebrates. Nonavian reptiles are underrepresented within the MHC literature and little is understood regarding the mechanisms maintaining MHC diversity in this vertebrate group. Here, we examined the relative roles of parasite‐mediated selection and sexual selection in maintaining MHC class I diversity of a color polymorphic lizard. We discovered evidence for parasite‐mediated selection acting via rare‐allele advantage or fluctuating selection as ectoparasite load was significantly lower in the presence of a specific MHC supertype (functional clustering of alleles): supertype four. Based on comparisons between ectoparasite prevalence and load, and assessment of the impact of ectoparasite load on host fitness, we suggest that supertype four confers quantitative resistance to ticks or an intracellular tickborne parasite. We found no evidence for MHC‐associated mating in terms of pair genetic distance, number of alleles, or specific supertypes. An association was uncovered between supertype four and male throat color morph. However, it is unlikely that male throat coloration acts as a signal of MHC genotype to conspecifics because we found no evidence to suggest that male throat coloration predicts male mating status. Overall, our results suggest that parasite‐mediated selection plays a role in maintaining MHC diversity in this population via rare‐allele advantage and/or fluctuating selection. Further work is required to determine whether sexual selection also plays a role in maintaining MHC diversity in agamid lizards.

## 1 INTRODUCTION

Pathogen–host relationships can strongly influence ecological and evolutionary processes within wild populations (Harvell, 2004). Understanding the mechanisms shaping host immunity is required for wildlife disease management and to evaluate the evolutionary consequences of disease (Acevedo‐Whitehouse & Cunningham, 2006). The major histocompatibility complex (MHC) is an extremely diverse gene family that plays a central role within the immune system of all jawed vertebrates (Kulski, Shiina, Anzai, Kohara, & Inoko, 2002). Both parasite‐mediated selection and sexual selection have been found to maintain this diversity (Piertney & Oliver, 2006). However, these alternative sources of selection are rarely examined together in the same species, limiting the ability to explore their relative roles (although see Dunn, Bollmer, Freeeman‐Gallant, & Whittingham, 2012; Eizaguirre et al., 2010; Sepil, Lachish, Hinks, & Sheldon, 2013; Sepil et al., 2015).

Evidence for parasite‐mediated selection acting on MHC genes via rare‐allele advantage (Borghans, Beltman, & De Boer, 2004; Schwensow et al., 2017), fluctuating selection (Jones, Cheviron, & Carling, 2015; Osborne, Pilger, Lusk, & Turner, 2017), heterozygote advantage (Doherty & Zinkernagel, 1975; Takahata & Nei, 1990), and optimal intermediate diversity advantage (Wegner, Kalbe, Kurtz, Reusch, & Milinski, 2003) has been uncovered across a range of taxa. However, most evidence points toward rare‐allele advantage and fluctuating selection as the dominant mechanisms by which pathogen‐mediated selection maintains MHC diversity, with little evidence that heterozygote advantage alone can account for the extreme diversity found at MHC loci (De Boer, Borghans, van Boven, Kesmir, & Weissing, 2004).

Individual MHC alleles or supertypes (functional clustering of alleles) may provide resistance against (Savage & Zamudio, 2011; Sepil et al., 2013), allow tolerance of (Regoes et al., 2014), or cause susceptibility to infection (Carrington et al., 1999). Interpreting the nature of such relationships requires information on both parasite prevalence and load, and the impact of infection on host fitness, data which are often difficult to obtain for populations in the wild (Råberg, 2014; Råberg, Sim, & Read, 2007). Resistance may come in the form of complete (qualitative) or partial (quantitative) protection against parasites (Westerdahl, Asghar, Hasselquist, & Bensch, 2011). Under qualitative resistance, the host prevents the establishment of infection and completely clears infection. Quantitative resistance, on the other hand, allows the host to suppress parasite load but not completely clear infection. Tolerance may co‐occur with quantitative resistance and refers to the ability of the host to withstand high parasite load without impacting fitness (Regoes et al., 2014). Tolerance is measured as the gradient of the relationship between Darwinian fitness (or a proxy of fitness) and infection intensity (Råberg, 2014). Finally, parasite counteradaptations to host defenses may cause certain MHC alleles or supertypes to increase host susceptibility to infection (Kubinak, Ruff, Hyzer, Slev, & Potts, 2012). Understanding the nature of host–parasite relationships is important as different types of relationships have different consequences for epidemiology and the evolutionary dynamics of both host and parasite (Westerdahl et al., 2011).

MHC‐associated mate choice has been discovered in most vertebrate classes, including bony fish (Evans, Dionne, Miller, & Bernatchez, 2012; Reusch, Häberli, Aeschlimann, & Milinski, 2001), amphibians (Bos, Williams, Gopurenko, Bulut, & DeWoody, 2009), reptiles (Miller, Moore, Nelson, & Daugherty, 2009; Olsson et al., 2003; Pearson, Godfrey, Schwensow, Bull, & Gardner, 2017), birds (Juola & Dearborn, 2012; Strandh et al., 2012), and mammals (Cutrera, Fanjul, & Zenuto, 2012; Schad, Dechmann, Voigt, & Sommer, 2012). Mate choice may be influenced by MHC diversity (high or intermediate), compatibility (high or intermediate diversity in offspring), and/or based on specific alleles or supertypes (Ejsmond, Radwan, & Wilson, 2014). Spatial or temporal differences in mate choice for MHC characteristics may also occur (fluctuating selection, Cutrera, Zenuto, & Lacey, 2014). In some systems, sexual selection may play a large role in maintaining MHC diversity. For instance, Winternitz et al. (2013) found that sexual selection explains more functional variation than parasite‐mediated selection in mammals.

Two nonmutually exclusive hypotheses are used to explain MHC‐associated mating: the good genes hypothesis and the complementary genes hypothesis. The good genes hypothesis involves mating that is influenced by MHC diversity, or specific alleles or supertypes, irrespective of the genotype of the choosy sex (absolute criteria, Brown, 1997; Hamilton & Zuk, 1982). The complementary genes hypothesis predicts that mating is based on MHC genotype compatibility between mates (self‐referential criteria, Zeh & Zeh, 1996). Hence, the genotype of the choosy sex is considered during mate choice. These hypotheses are used to test for evidence of heterozygote or intermediate diversity advantage, or associations with certain alleles or supertypes, indicating rare‐allele advantage or fluctuating selection (Spurgin & Richardson, 2010). Both olfactory (Boehm & Zufall, 2006; Milinski et al., 2005; Setchell et al., 2011; Strandh et al., 2012) and visual (Dunn et al., 2012; Hinz, Gebhardt, Hartmann, Sigman, & Gerlach, 2012; Milinski, 2014; Olsson et al., 2005) traits have been proposed to signal individual MHC genotypes to conspecifics in mammals, birds, and fish. For instance, Dunn et al. (2012) found that the male black facial masks of common yellowthroat birds likely act as a signal of MHC diversity to mates, and MHC‐dependent peptides in mouse urine may signal MHC genotype to conspecifics (Sturm et al., 2013). However, the phenotypic traits used by reptiles and amphibians to signal MHC genotype to conspecifics are largely unknown.

Here, we examined the relative roles of sexual selection and parasite‐mediated selection in maintaining MHC diversity within a wild reptile population. The Australian tawny dragon lizard (*Ctenophorus decresii*), for which MHC class I has been characterized (Hacking, Bertozzi, Moussalli, Bradford, & Gardner, 2018), is host to both ectoparasites and intracellular parasites (Hacking et al., unpublished data). Male *C. decresii* exhibit secondary sexual coloration on their throat and chest that is emphasized in displays to conspecifics (Gibbons, 1979; Osborne, 2005a,b; Osborne, Umbers, Backwell, & Keogh, 2012; Stuart‐Fox & Johnston, 2005). Furthermore, in some populations four discrete male throat color morphs coexist. Hence, *C. decresii* represents an excellent model to investigate patterns of MHC variation, parasites, and visual signals. First, we investigated the role that parasite‐mediated selection plays in maintaining MHC diversity by testing the hypothesis that specific MHC supertypes are associated with parasite prevalence and/or load. We then determined whether MHC–parasite relationships were associated with resistance, tolerance, or susceptibility. Second, we asked whether sexual selection, via MHC‐associated mating, plays a role in maintaining MHC diversity. In a specific manner, we tested the hypothesis that MHC diversity and/or mate MHC compatibility predicts male mating status while accounting for the spatial position of mates, pair relatedness, and mate overall genetic diversity. Finally, we investigated visual phenotypic traits that may signal MHC genotype to conspecifics.

## 2 MATERIALS AND METHODS

### 2.1 Male mating status

The tawny dragon is a small (<30 g) agamid lizard that is endemic to the rocky ranges of South Australia. Individuals were captured from a site near Hawker in the Flinders Ranges, South Australia (31°57′17.5″S, 138°22′26.4″E) by noosing and were then released at the point of capture. We sampled individuals during spring and summer between 2013 and 2015 (two seasons), and captive hatching was undertaken during spring and early summer of 2014, with each mother sampled once within the breeding season. We focussed on sampling adult males within the population and only sampled gravid females for captive hatching. Captive hatching and subsequent paternity analysis presented in Hacking, Stuart‐Fox, and Gardner (2018) produced 21 complete family groups, within which there was no evidence for multiple paternity. The tawny dragon employs a mostly polygynous genetic mating system (Hacking, Stuart‐Fox et al., 2018), with males patrolling territories of 213 m^2^, on average (Yewers, 2016). Male territories likely contain the home ranges of several females, and a female's home range could overlap the territory of more than one male.

When investigating mate choice, it is important to take the spatial position of potential mates into account as females are likely to only come into contact with males that are close‐by. Failing to account for the geographic distance between females and potential mates may cause mating preferences to be missed. We recorded the location (±3 m) of each individual at capture using a Garmin^©^ handheld GPS. For each mother, adult males that were captured within a 100 m radius were considered to be potential mates. Given that male territory size ranges from 1 to 898 m^2^ and averages 213 m^2^ (Yewers, 2016), this 100 m radius is large enough to encompass most males that a female may come into contact with but may also include some males that a female did not come into contact with. The average number of males available to each female was 21 (range 9–36). Four males mated with more than one female, and most “mated” males were available to other females during the breeding season. The geographic distance between all pairs (mated and available) was calculated.

### 2.2 Parasite infection

We recorded the number of attached ticks (*Amblyomma limbatum*) for each male (Supporting Information Figure S1) captured between 2013 and 2015 (137 total). Because we focussed on sampling only adult males, females are not included in this dataset. Associations between the MHC and arachnid ectoparasites have been uncovered in both mammals (Kamath, Turner, Kusters, & Getz, 2014; Oliver, Telfer, & Piertney, 2009; Schad et al., 2012) and reptiles (Radwan, Kuduk, Levy, LeBas, & Babik, 2014). Immune defense against hematophageous ectoparasites involves class II MHC molecules and likely also MHC class I molecules via cross‐presentation (Andrade, Teixeira, Barral, & Barral‐Netto, 2005; Rock, Reits, & Neefjes, 2016; Wikel, 1996). Furthermore, arachnid ectoparasites (ticks and mites) have been found to transmit vectorborne intracellular parasites such as protists, viruses, and bacteria to reptile hosts (Allison & Desser, 1981; Bonorris & Ball, 1955; Camin, 1948; Chaisiri, McGarry, Morand, & Makepeace, 2015; Reynolds, Hart, Hermance, Brining, & Thangamani, 2017; Smallridge & Bull, 1999). In fact, *A. limbatum* is known to transmit haemogregarine blood parasites to an Australian skink species, *Tiliqua rugosa* (Smallridge & Bull, 1999). MHC I molecules play a direct role in immune defense against such intracellular parasites (Janeway, Travers, Walport, & Shlomchik, 2005). Vectorborne parasite infections in hosts are often influenced by host vector load (Bennett & Cameron, 1974; Godfrey, Nelson, & Bull, 2011; Reardon & Norbury, 2004; Sol, Jovani, & Torres, 2000). For instance, Godfrey et al. (2011) found that higher tick numbers on hosts were consistently associated with higher tickborne haemogregarine blood parasite load and prevalence in Tuatara, *Sphenodon punctatus*.

### 2.3 Male morphometrics and coloration

**Figure 1:**
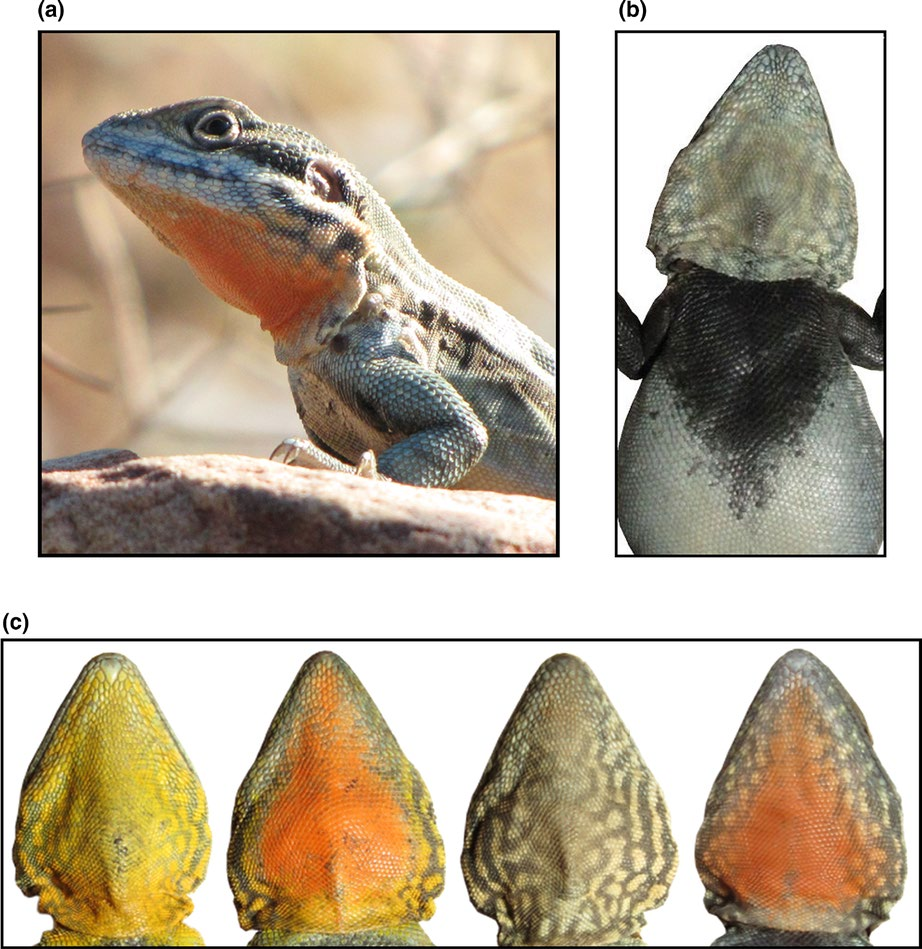
A male tawny dragon lizard (*Ctenophorus decresii*) at the Hawker field site in the Flinders Ranges, South Australia (a). An example of an adult male chest patch marking (b), and the four throat color morphs present within the Hawker population, ordered left to right from most frequent to least frequent (c)

Snout‐to‐vent length (SVL) was measured for each male to the nearest 0.01 mm and mass to the nearest 0.25 g. Male body condition was estimated using residuals from a regression of adult male mass and SVL. Male *C. decresii* exhibit polymorphism for throat color within the species’ northern lineage, which includes the focal population (McLean, Stuart‐Fox, & Moussalli, 2014; Teasdale, Stevens, & Stuart‐Fox, 2013). Four discrete, heritable morph types exist within these populations; orange surrounded by yellow (orange/yellow), orange, yellow, or gray (McLean et al., 2014; Teasdale et al., 2013; Figure 1). The amount of color on the throat and throat brightness is highly variable within morph types, which is likely influenced by both genetic and environmental factors (Rankin, McLean, Kemp, & Stuart‐Fox, 2016; Teasdale et al., 2013). For instance, throat brightness and the amount of gray color (associated with melanin pigment) are influenced by stress hormones under laboratory conditions (Lewis, Rankin, Pask, & Stuart‐Fox, 2017), and carotenoid pigmentation can only be obtained from the diet (McGraw, 2006). The throat is emphasized during male displays (Gibbons, 1979), and it is likely that throat coloration plays an important role in social signaling (Yewers et al., 2016). Morph types do not differ in regard to morphology or microhabitat use (Teasdale et al., 2013; Yewers, 2016). To capture variation in color within and among throat morphs, we calculated the proportion of orange and yellow on the throat of each male. Variation in achromatic throat coloration (‘brightness’) among males, which is independent of morph type (Teasdale et al., 2013), was also calculated for each male. Male *C. decresii* also possess gray to black (melanin) chest patch markings that are exposed during male displays (Gibbons, 1979; Figure 1). Chest patch size is an important signal in male–male interactions (Osborne, 2005a), and in the congener *C. Ornatus,* chest patch size is associated with territory size and the number of females within a male's territory (Lebas, 2001). We therefore also calculated male relative chest patch size. Refer to Supporting Information (Data S1, Figure S2) for additional details on the quantification of male throat coloration and chest patch size.

### 2.4 MHC genotyping and supertype analysis

MHC genotypes were obtained using next‐generation amplicon sequencing and a thorough genotyping protocol, Hacking, J., Bradford, T., Pierce, K. and Gardner, M., unpublished data. The α1 domain (exon 2), which forms part of the peptide‐binding cleft of the MHC I molecule was targeted to gain information on the functional diversity present. As discussed in Hacking, J., Bradford, T., Pierce, K. and Gardner, M. (unpublished data), the MHC I α1 alleles used here likely represent a subsample of the diversity present at the *C. decresii* MHC I region, as is likely the case for many other MHC studies on nonmodel organisms (Babik, 2010).

It is the characteristics of peptide‐binding sites and other important sites, such as those involved in T‐cell receptor interactions within the α1 and α2 domains that form the binding cleft of MHC molecules that determine associations between MHC molecules and parasite‐derived peptides. Such sites are likely to be under positive selection. Clustering MHC alleles into functional groups (supertypes) based on the properties of positively selected sites (PSS) and putative peptide‐binding sites allows the phenotypic effects of MHC alleles to be examined (Naugler & Liwski, 2008; Trachtenberg et al., 2003). Accordingly, PSS within the α1 domain identified in Hacking, J., Bradford, T., Pierce, K. and Gardner, M., (unpublished data) were used to cluster *C. decresii* alleles (including all populations sampled in Hacking, J., Bradford, T., Pierce, K. and Gardner, M., (unpublished data)) into supertypes based on five amino acid physicochemical descriptors: hydrophobicity, steric bulk, polarity, and two electronic effect variables (Doytchinova & Flower, 2005; Sandberg, Eriksson, Jonsson, Sjostrom, & Wold, 1998). First, amino acid positions under positive selection (*n* = 9) were extracted from the rest of the sequence and the physicochemical properties (Doytchinova & Flower, 2005; Sandberg et al., 1998) of each amino acid for each allele were recorded. These data were formatted as a matrix with alleles in rows and the physicochemical properties of each amino acid as columns. Alleles were then clustered into supertypes using K‐means clustering, implemented using the *adegenet* package (Jombart, 2008) in R ver. 3.4.1 (R Core Team 2016). For K‐means clustering, all principal components were retained (*n* = 25) and the optimal number of clusters was determined based on Bayesian information criterion (BIC) (Jombart, Devillard, & Balloux, 2010).

### 2.5 Pair relatedness and genomewide diversity

Under MHC‐associated mate choice, a lack of correlation between MHC diversity and genomewide diversity would suggest that mating is not simply due to mate choice for genetically diverse individuals, while a lack of correlation between relatedness and MHC similarity eliminates mating due to general inbreeding avoidance. Therefore, we tested for correlations between MHC diversity (number of MHC alleles) and an estimate of genomewide diversity, and between‐relatedness estimates and percent genetic (amino acid) distance among MHC I alleles shared between pairs. Microsatellite genotypes presented in Hacking, Stuart‐Fox et al. (2018) were used to estimate relatedness between each mother, her mate, and all available males, using Coancestry ver. 1.0.1.5 (Wang, 2007). Microsatellite genotypes were also used to estimate individual heterozygosity using the *genhet* package (Coulon, 2010) in R (R Core Team 2016) as a measure of neutral genomewide diversity. Correlation analyses were undertaken using the *glm* function in R (R Core Team 2016). For both relatedness and genomewide diversity estimates, eight microsatellite loci were used, seven of which have high polymorphic information content values (≥0.85) (Hacking, Stuart‐Fox, et al., 2018). Note, however, that it is difficult to gain an accurate estimation of genomewide diversity and these microsatellite loci may not be adequate (DeWoody & DeWoody, 2005; Väli, Einarsson, Waits, & Ellegren, 2008), although see (Ljungqvist, Akesson, & Hansson, 2010).

### 2.6 Hypothesis testing overview

An AICc‐based information‐theoretic approach was used to test our alternative hypotheses about the factors driving MHC diversity in this system (Burnham & Anderson, 2004; Burnham, Anderson, & Huyvaert, 2011; Galipaud, Gillingham, David, & Dechaume‐Moncharmont, 2014; Grueber, Nakagawa, Laws, & Jamieson, 2011; Symonds & Moussalli, 2011). We examined evidence for parasite‐mediated selection in model set 1 and MHC‐associated mating in model set 2. We then investigated potential phenotypic signals of MHC genotype in model set 3. Correlations between predictor variables were investigated, and variance inflation factors were calculated (VIF, *usdm* R package, Naimi, Hamm, Groen, Skidmore, & Toxopeus, 2014) to avoid multicollinearity within models. A VIF below 3 was considered acceptable (Zuur, Ieno, & Elphick, 2010). See Supporting Information Table S1 for an outline of all models.

We used generalized linear models (GLMs) or generalized linear mixed models (GLMMs) to estimate the effect of potential predictors on response variables that were normally distributed. Three response variables, percentage of throat colored orange, percentage of throat colored yellow, and tick load, were zero‐inflated with “true zeros” or “structure zeros”; that is, zeros resulting from subpopulations within the dataset rather than from random sampling (‘false zeros’ or ‘sampling zeros’, He, Tang, Wang, & Crits‐Christoph, 2014; Martin et al., 2005). For example, the percentage of throat colored orange variable is a measure of the percentage of a male's throat colored orange and is zero‐inflated because two of the four morph types (gray and yellow) do not include orange coloration. Due to the nature of the zero‐inflation, hurdle models were fitted when percentage of throat colored orange, percentage of throat colored yellow, and tick load were used as response variables. Hurdle models include two components: First, a binomial model determines whether a zero or count (nonzero) outcome occurs (presence/absence). Second, a zero‐truncated (excluding zeros) model (e.g., Poisson) analyses the count data (Dalrymple, Hudson, & Ford, 2003; Guo et al., 2016; Hassrick et al., 2016; Naimi et al., 2014; Welsh, Cunningham, Donnelly, & Lindenmayer, 1996; Xu, Paterson, Turpin, & Xu, 2015). The fit of both a negative binomial and Poisson distribution was considered for the zero‐truncated (count) part of the hurdle models.

For each model set, a global model was constructed, which was standardized using the *arm* R package (Gelman, 2008). Standardization allowed direct comparison among predictor variables during model selection (Gelman, 2008; Grueber et al., 2011). The *lme4* R package was used to construct GLMMs (Bates, Mächler, Bolker, & Walker, 2015), the *stats* R package was used to construct GLMs (R Core Team 2016), and the *pscl* R package was used to construct hurdle models (Jackman, 2012; Zeileis, Kleiber, & Jackman, 2008). Then, the *dredge* function from the *MuMIn* R package (Bartoń, 2009) was used to construct all possible models based on the global model, including the null model. When covariates were used in models, they were included in all models, including the null model, constructed by the *dredge* function. The top models were extracted based on a ΔAICc 95% confidence set (Symonds & Moussalli, 2011). At last, top models were averaged so that parameters were recalculated based on the top model set alone. Model selection and averaging were undertaken using the *MuMIn* R package (Bartoń, 2009). Model fit was examined using adjusted *R* ^2^ for GLMs (Nagelkerke, 1991) and marginal ($R_{m}^{2}$) and conditional ($R_{c}^{2}$) *R* ^2^ for GLMMs (Nakagawa & Schielzeth, 2013). *R* ^2^ calculations were undertaken using the *MuMIn* R package (Bartoń, 2009). The fit of hurdle models was visually assessed using rootograms, which were created using the *countreg* R package (Kleiber & Zeileis, 2016).

The support for models within model sets was determined based on ΔAICc, the evidence ratio (how much better one model explains the data than the next model) and model fit. The importance of specific predictor variables was based on effect size, accumulative Akaike weights (relative importance), and statistical significance (based on 95% CIs).

### 2.7 Hypothesis testing: parasite‐mediated selection

In model set 1, we tested the hypothesis that the presence of specific MHC I supertypes predicts tick prevalence and/or load, using a hurdle model (Supporting Information Table S1). Such a relationship would indicate that parasite‐mediated selection may occur via rare‐allele advantage or fluctuating selection (Spurgin & Richardson, 2010). We could not estimate MHC heterozygosity because we amplified MHC I alleles across multiple loci (up to four alleles per individual, Hacking, J., Bradford, T., Pierce, K. and Gardner, M., unpublished data) and therefore did not test for evidence of parasite‐mediated selection acting through heterozygote advantage (Spurgin & Richardson, 2010). As tick load varied by year and with time in season (early vs. late), these variables were included as covariates in model set 1. Tick load was not correlated with individual absolute mass; therefore, mass was not used as a covariate.

Information on the impact of parasite load on host fitness is required when delineating MHC–pathogen relationships (Råberg et al., 2007). Body condition was used as a proxy for fitness and was calculated using residuals of a regression of SVL against mass (Jakob, Marshall, & Uetz, 1996). We plotted male body condition against tick load and grouped data based on the presence and absence of MHC I supertypes that were identified in model selection. A linear regression line was fitted for each group (absence/presence) for each supertype to assess the relationship between body condition and tick load using the *stats* R package (R Core Team 2016).

### 2.8 Hypothesis testing: sexual selection

We tested two alternative but potentially nonmutually exclusive hypotheses for MHC‐associated mating; (a) “mate choice” for MHC diversity or specific MHC supertypes; the good genes hypothesis*,* and (b) “mate choice” for MHC‐compatible individuals; the complementary genes hypothesis (Supporting Information Table S1, Eizaguirre, Yeates, Lenz, Kalbe, & Milinski, 2009; Landry, Garant, Duchesne, & Bernatchez, 2001; Miller et al., 2009; Pearson et al., 2017; Sepil et al., 2015). Mate choice was determined by comparing the male that a female mated with to a set of “available” males (within a 100 m radius). This variable may reflect female mate choice (intersexual selection) and/or male–male competition for access to females (intrasexual selection) but is not a measure of male choice or male reproductive success as all females available to males were not sampled. For model set 2, MHC diversity (good genes hypothesis) was estimated as the number of male MHC I alleles and pair MHC I genetic distance (complementary genes hypotheses) was estimated as the average percent genetic distance (amino acid) between shared MHC I alleles of a male and female pair. Male mass and the spatial proximity (m) of available and mated males were included as covariates in model set 2 because these were strong predictors of male mating status in preliminary analyses, with mated males larger, and geographically closer, than available males. Male and female ID were included as random factors within the model to account for repeated individuals within the dataset (i.e., many males were available to a single female, some males mated with more than one female, and many males were both mated and available to other females). To test whether the probability of possessing a particular supertype is dependent on male mating status (good genes hypothesis), we performed a Fisher's exact test, implemented in R using the *stats* package (R Core Team 2016) and the *rcompanion* package (Mangiafico, 2015).

Following model set 2, we investigated potential phenotypic (coloration) signals of male MHC diversity, including all adult males sampled within the population (*n* = 108, model set 3a‐f, Supporting Information Table S1). We included percentage of throat colored orange, percentage of throat colored yellow, throat brightness, and chest patch as predictors. Male number of alleles (model set 3a) and male number of supertypes (model set 3b) are measures of MHC diversity and were used as response variables. We also tested for associations between potential phenotypic signals and specific MHC I supertypes (model sets 3c–3f). Each of the potential phenotypic signals was used as a response variable, and each supertype was included as a separate predictor variable, coded as present or absent for each individual. To further investigate trends uncovered between throat color and supertype four (model sets 3c and 3d), we performed a Fisher's exact test to determine whether the probability of possessing supertype four is dependent on male throat morph type (yellow/orange‐yellow/orange/gray).

To confirm that the phenotypic traits that were found to be associated with specific supertypes also predicted mating status, we performed a GLM with mating status as the response and male percentage throat colored yellow and male percentage throat colored yellow as predictors (model set 3g, Supporting Information Table S1). As with model set 2, male mass was used as a covariate. Only males were included in the mating status variable, rather than pairs of males and females. We also performed a Fisher's exact test to determine whether mating status is dependent upon male throat morph type.

## 3 RESULTS

### 3.1 MHC genotyping and supertype analysis

Most of the mothers (18/21, 90%), fathers (14/16, 88%), and available males (95/108, 88%), and 86% (144/166) of the individuals used to test for parasite‐mediated selection were successfully genotyped for the MHC I α1 domain. Individuals had between one and four alleles and pair MHC I genetic distance varied from 0.02 to 0.36. A total of 28 MHC I alleles were uncovered, which translated into 27 unique amino acid sequences (Hacking, J., Bradford, T., Pierce, K. and Gardner, M., unpublished data). The number of alleles per individual, as measured by nucleotide sequences, was the same as when measured by amino acid sequences.

Alleles from all *C. decresii* populations sampled in Hacking, J., Bradford, T., Pierce, K. and Gardner, M., (unpublished data) clustered into eight supertypes (Supporting Information Figure S3, S4, and S5), and individuals had between one and four supertypes. Seven of these supertypes (ST2 – ST8) were present within the focal population. Most (89%) individuals with more than one MHC allele had an equal number of alleles and supertypes, indicating high within‐individual functional diversity. The frequency of each supertype varied, with supertype three present in 52% of individuals and supertype seven present in only 4% of individuals. Supertype six was also rare (9%), and all other supertypes had an intermediate frequency (18%–24%, Supporting Information Figure S5).

### 3.2 Pair relatedness and genomewide diversity

There was no correlation between number of male MHC I alleles and microsatellite heterozygosity (*R* ^2^ = 0.002, *p* = 0.193) or between MHC I genetic (amino acid) similarity between pairs and pair relatedness (*R* ^2^ = 0.001, *p* = 0.240, Supporting Information Figure S6). This suggests that it is not likely that MHC‐associated mating patterns observed are simply due to mate choice for genetically diverse individuals or mating to avoid inbreeding.

### 3.3 Parasite‐mediated selection

**Figure 2:**
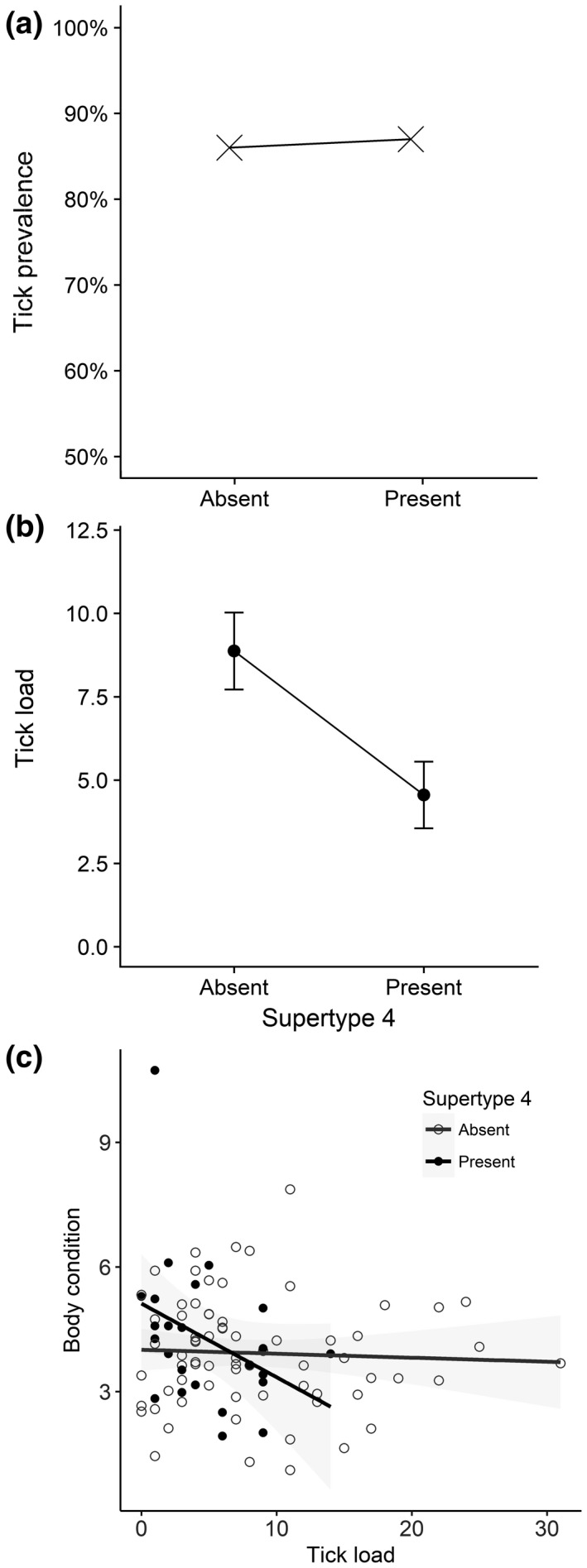
Relationship between the presence and absence of supertype four and tick prevalence (a) and mean (±*SE*) tick load (b), and the relationship between tick load and body condition (showing 95% confidence intervals with shaded area and dotted lines), comparing individuals with and without supertype four (c)

Overall, 95% of individuals were infected with ticks and average tick load was eight. We uncovered an association between a certain supertype and tick load. The top model included only supertype four, which was present in all models with a ΔAICc less than two. Supertype three was also in models with ΔAICc less than two (Table 1). These associations were driven by the count component of the hurdle model, suggesting that the presence or absence of these supertypes is associated with the tick load, rather than tick prevalence. Indeed, tick prevalence is similar in the presence and absence of supertype four (Figure 2a). Tick load was only statistically significantly different in the respect to supertype four (95% CIs ‐0.98, ‐0.15; odds ratio 0.57), with mean tick load lower in the presence of this supertype (Figure 2b). The rootogram confirmed that the hurdle model provided a good fit for the data (Supporting Information Figure S7).

**Table 1:** AIC information‐theoretic top model selection results for model set 1 (response: tick load, predictors: MHC I supertypes), after model averaging. Only those models with ΔAICc ≤ 2 are shown due to the large number of models in the 95% confidence set. See Supporting Information Table S3 and Figure S10 for summary results for variables and Supporting Information Figure S7 for overall model fit

Model	*df*	AICc	ΔAICc	Weight	ER
ST4	9	751.81	0	0.19	
ST4 + ST3	11	753.41	1.60	0.09	2.1

Note

ER: evidence ratio.

The significant negative relationship between supertype four and tick load, and the neutral relationship between supertype four and tick prevalence, suggests that this supertype confers quantitative resistance to ticks or a tickborne parasite. To investigate whether supertype four also plays a role in tolerance of ticks (or a tickborne parasite), we examined the relationship between body condition (a measure of fitness) and tick load in light of the presence and absence of supertype four (Figure 2c). When only individuals that do not possess supertype four are considered, there is a slight nonsignificant negative relationship (*p*‐value = 0.69, slope = −0.01, Figure 2c) between body condition and tick load, indicating that high tick loads probably have a small impact on body condition. In contrast, individuals that possess supertype four show a much steeper decline in body condition with increasing tick load, although this relationship is also not statistically significant (*p*‐value = 0.08, slope = −0.18, Figure 2c). It is therefore likely that supertype four offers only resistance and not tolerance to ticks or a tickborne parasite.

### 3.4 MHC‐associated mating

**Figure 3:**
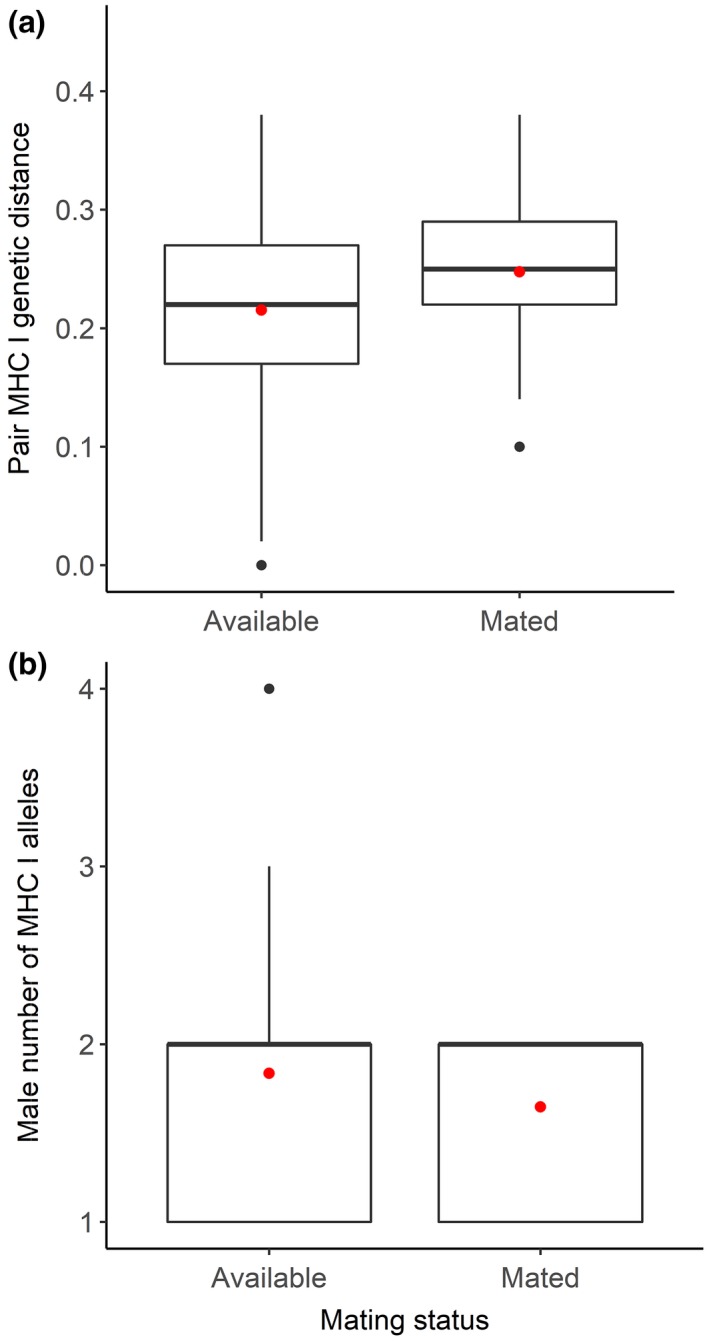
Difference between available and mated *Ctenophorus decresii* males in average pair percent difference among shared MHC I alleles (a) and male number of MHC I alleles (b). Mean values are indicated with a red point

We found no evidence for mate choice based on MHC diversity or specific MHC supertypes (good genes hypothesis) or pair MHC I genetic distance (complementary genes hypothesis). Both pair MHC I genetic distance and male number of alleles were not significantly different between mated and available pairs (Figure 3), and the probability of possessing a certain supertype was independent of male mating status. Refer to Supporting Information for details of MHC‐associated mating results (Data S2).

### 3.5 Potential signals of MHC genotype

**Figure 4:**
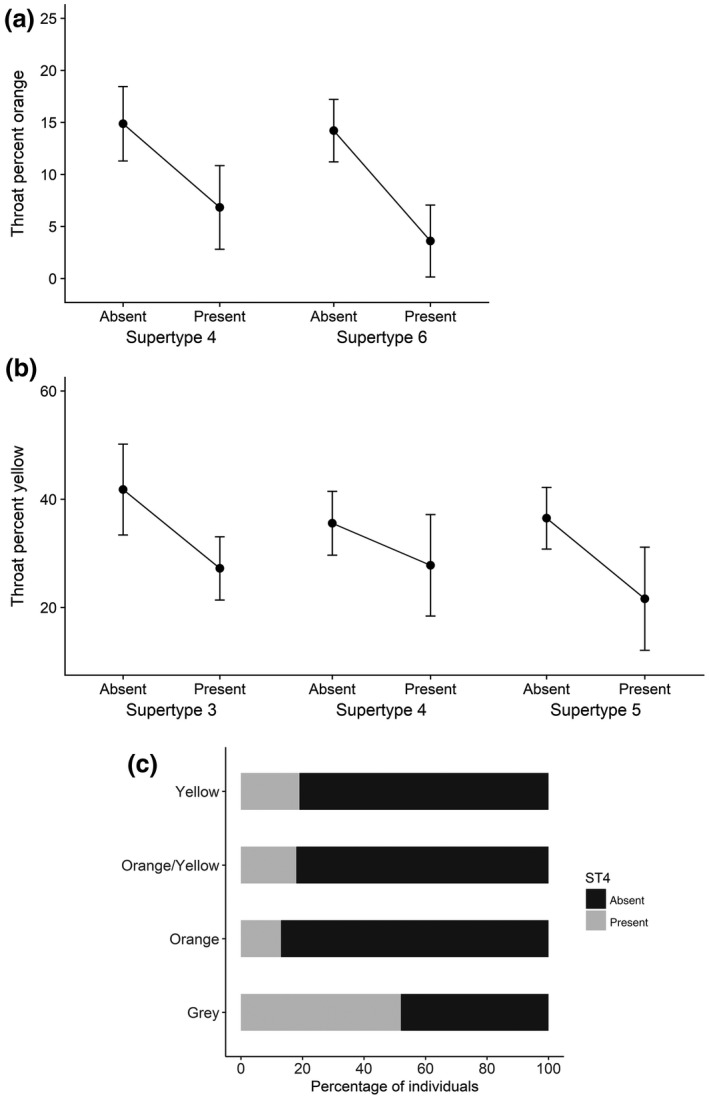
Mean (±*SE*) percentage of male throat color (a: orange, b: yellow) as a function of supertype presence (only supertypes that were present in models with ΔAICc ≤ 2 are displayed) and the percentage of individuals of each male throat morph that possess supertype four (c)

We found little evidence to suggest that male coloration signals MHC genotype to conspecifics. Neither the number of alleles nor the number of supertypes was associated with male coloration (throat color, throat brightness, and relative chest patch size). Based on model sets 3c‐f, there were no significant associations between male coloration and the presence of certain supertypes (Figure 4). Refer to Supporting Information for details of model set 3a–3f results (Data S3). However, it was observed that supertype four is less likely to occur in orange, orange/yellow, and yellow morphs. Indeed, Fisher's exact test revealed that the probability of possessing supertype four is dependent upon morph type (*p*‐value = 0.02), with over 50% of gray morphs possessed supertype four, whereas <20% of yellow, orange, and orange/yellow morphs possessed this supertype (Figure 4c).

Because we uncovered trends suggesting that percentage of throat colored yellow and percentage of throat colored orange may be associated with certain MHC I supertypes, we investigated whether these variables predicted mating status (model set 3g). The null model had the lowest ΔAICc and 95% CIs for percentage of throat colored yellow and percentage of throat colored orange overlapped zero, suggesting that percentage of throat colored orange and percentage of throat colored yellow are not strong predictors of mating status (Supporting Information Tables S18 and S19Figures S8 and S9). Furthermore, a Fisher's exact test revealed that male mating status is independent of male throat morph type (*p*‐value = 0.58).

## 4 DISCUSSION

We tested hypotheses regarding the roles of parasite‐mediated selection and sexual selection in maintaining diversity at the MHC within a wild agamid lizard population. The roles of both parasite‐mediated selection and sexual selection are rarely considered together in a single population, limiting the ability to make inferences about the relative importance of these mechanisms. Results supported the hypothesis that specific MHC I supertypes are associated with parasite infection, indicating a role for parasite‐mediated rare‐allele advantage and/or fluctuating selection in maintaining MHC diversity. Parasite load was significantly lower in the presence of MHC I supertype four, but this supertype had no effect on parasite prevalence, indicating quantitative resistance. Furthermore, the relationship between parasite load and body condition in the presence of supertype four indicated that this supertype offers only resistance, not tolerance. Tolerance is rarely considered when delineating host–parasite relationships despite important implications for epidemiology and host–parasite coevolutionary dynamics.

In contrast to parasite‐mediated selection, we found no evidence that sexual selection plays a role in maintaining *C. decresii* MHC I diversity. There was little support for mating based on MHC diversity, specific MHC supertypes, or pair MHC compatibility when taking potential mate spatial proximity, inbreeding avoidance, and overall genetic diversity into account. Similar results have recently been reported for great tit birds, in which there is strong evidence for parasite‐mediated selection (Sepil et al., 2013) but little evidence for sexual selection (Sepil et al., 2015) maintaining MHC diversity. In line with the lack of evidence for MHC‐associated mating, we did not find any evidence to suggest that male coloration acts as a signal of MHC genotype to conspecifics. However, we did find an association between male throat color morph and supertype four, with gray‐throated males over twice as likely to possess this supertype compared to other morphs. The higher likelihood of possessing supertype four may help to maintain this morph within the population. Links between the MHC and coloration have been found in other systems, although the mechanisms underlying these associations are yet to be elucidated.

### 4.1 Parasite‐mediated selection

Understanding the genetic basis of variation in infections within and among wildlife populations requires identification of host–parasite immunogenetic relationships (i.e., resistance, tolerance, and susceptibility) and the evolutionary mechanisms driving such relationships. In recent times, researchers have aimed to better understand the relationships between host genetic immunity and parasites, attempting to differentiate qualitative resistance, quantitative resistance, and susceptibility. For instance, Sepil et al. (2013) discovered that two different MHC supertypes are associated with two different avian malaria (*Plasmodium*) species, but one confers qualitative resistance and the other offers quantitative resistance. However, the role of host tolerance is rarely considered in such studies. One of the few examples of research on the role that MHC molecules play in tolerance is Regoes et al. (2014), who found that MHC heterozygosity is associated with greater tolerance of human HIV infections.

Our results suggest that supertype four confers quantitative resistance against but not tolerance of ticks or a tickborne parasite infecting *C. decresii*. Consistent with resistance, we uncovered a negative relationship between supertype four and parasite load. Associations between infection prevalence and specific MHC alleles or supertypes are likely linked to the ability to completely clear infection, not the ability to prevent infection. This is because the MHC is not directly involved in the initial innate immune response to infection (Chaplin, 2010). We uncovered a neutral relationship between supertype four and parasite prevalence, indicating that this supertype does not play a role in clearing infection, as expected under quantitative resistance.

It is likely that supertype four does not offer tolerance alongside quantitative resistance, as revealed by the negative relationship between body condition and parasite load in the presence of this supertype. In the absence of supertype four body condition decreased with an increase in parasite load at a lower rate. Discrepancy between individuals with and without supertype four in regard to the tolerance gradient may be caused by a cost associated with immune response. For instance, immune response is negatively correlated with reproductive effort in birds (Knowles, Nakagawa, & Sheldon, 2009) and with body size and development time in field crickets (Rantala & Roff, 2005). However, often multiple measures of fitness are required to gauge the impact of infection on individuals. Furthermore, it is unknown whether body condition correlates with reproductive success in *C. decresii* or agamid lizards more generally. Recent work in anolis lizards (Cox & Calsbeek, 2015) suggests that body condition may not be an accurate measure of fitness for lizards. Hence, the decrease in body condition associated with increasing parasite load observed for *C. decresii* may not necessarily indicate an impact of parasite load on lizard health or relative fitness.

When testing the parasite‐mediated selection hypothesis, we did not take into account risk of infection. Individuals that are unexposed to ticks cannot become infected regardless of their MHC genotype. For instance, sleepy lizards (*Tiliqua rugosa*) that are highly socially connected and use the same refuges as neighboring lizards have higher tick loads (Leu, Kappeler, & Bull, 2010). Therefore, if variation in tick exposure exists within the *C. decresii* population the relationship we observed between specific supertypes and tick prevalence may be biased. At last, it is worth noting that in this study we only considered the α1 domain from a subset of *C. decresii* MHC I loci. We did not include the α2 domain, which together with α1, completes the peptide‐binding region, or MHC class II loci. Future studies will likely benefit from including the entire peptide‐binding region or complete MHC haplotypes.

We identified an association between a specific MHC genotype (supertype four) and parasite load, which is one of the signatures expected under parasite‐mediated rare‐allele advantage and fluctuating selection (Spurgin & Richardson, 2010). Further work involving long‐term spatiotemporal data is required to assess whether rare‐allele advantage or fluctuating selection play a dominant role, or whether they are acting together in this system. Furthermore, we were unable to test for heterozygote advantage because alleles were amplified across multiple loci. Therefore, it is possible that parasite‐mediated heterozygote advantage also plays a role in maintaining MHC diversity in *C. decresii*.

### 4.2 Sexual selection: MHC‐associated mating

We uncovered limited evidence for MHC‐associated mating in regard to both the good genes and complementary genes hypotheses. This suggests that MHC I loci play a secondary role or are of little importance in mate choice decisions in *C. decresii*. In contrast to our results, the few studies that have examined MHC‐associated mate choice in nonavian reptiles have uncovered significant relationships between measures of MHC diversity or dissimilarity and mating (Miller et al., 2009; Olsson et al., 2005; Pearson et al., 2017). Other studies have reported a lack of evidence for MHC‐associated mating (Kuduk et al., 2014; Whitcomb, Banks, & O'Malley, 2014). For instance, Sepil et al. (2015) found no evidence for MHC‐disassortative mating in wild great tits despite large sample sizes. Moreover, we did not identify male phenotypic signals of MHC genotype, providing further evidence that class I MHC may play little role in mate choice. However, it is possible that other phenotypic traits that we did not measure, such as femoral pore secretions or other olfactory cues signal MHC I genotype to conspecifics. It is important to note that the sample size associated with the mating status variable may have inhibited our ability to identify small effects related to MHC‐associated mating and phenotypic mating signals. Furthermore, we only considered a single exon from a subset of MHC I loci; MHC‐associated mate choice may be detected when entire MHC I haplotypes or MHC class II loci are considered.

### 4.3 Supertype four, parasites, and male morph type

Supertype four likely provides quantitative resistance to ticks or a tickborne parasite in *C. decresii*. This supertype was also associated with male throat color; the gray morph was more likely to possess supertype four compared to other morphs. The gray morph is the least bold and least aggressive and has low testosterone levels compared to the three other male morphs (Yewers, Jessop, & Stuart‐Fox, 2017; Yewers et al., 2016). Despite these characteristics likely reducing the ability of gray morphs to defend territories and obtain mates, the gray morph is present within all polymorphic populations, implying a compensatory selective advantage (McLean et al., 2014). Perhaps a superior ability to reduce parasite load could provide the gray morph with a selective advantage, allowing it to be maintained within the population. Lehnert, Pitcher, Devlin, and Heath (2016) describe a similar scenario in Chinook salmon (*Oncorhynchus tshawytscha*), in which heritable morphs differ significantly in regard to MHC class I and II variables. Compared to other morphs, the gray morph has the highest expression levels of several genes associated with melanin synthesis (McLean, Lutz, Rankin, Stuart‐Fox, & Moussalli, 2017). Melanin coloration has also been linked to the MHC in the common yellowthroat, *Geothlypis trichas* (Dunn et al., 2012) and brown trout, *Salmo trutta* (Jacob, Evanno, Von Siebenthal, Grossen, & Wedekind, 2010). Although the genetic and biochemical pathways responsible for this link are unknown, melanins undertake several functions within the immune system (Nosanchuk & Casadevall, 2006), suggesting a possible link between MHC molecules and melanin pigmentation.

## 5 CONCLUSION

Both parasite‐mediated selection and sexual selection are rarely studied together in a single population when investigating the mechanisms maintaining MHC diversity. Here, we considered both sources of selection and discovered evidence for parasite‐mediated selection but little evidence for MHC‐associated mate choice. When testing MHC‐associated mate choice hypotheses, we controlled for spatial proximity among individuals, relatedness, and overall genetic diversity. Such potential confounding variables are not always considered in studies examining mate choice for MHC characteristics. Our results suggest that parasite‐mediated selection may be acting via rare‐allele advantage and/or fluctuating selection to maintain MHC diversity with *C. decresii*, with a specific supertype likely conferring quantitative resistance to ticks or a tickborne parasite. Overall, this study supports a dominant role for parasite‐mediated selection in maintaining MHC diversity and further demonstrates that the role that sexual selection plays is highly variable within and among vertebrate groups.

## AUTHOR CONTRIBUTIONS

J.H., D.S‐F., and M.G. designed research, and J.H. performed research, analyzed data, and wrote the manuscript. D.S‐F., S.G., and M.G. contributed toward writing the manuscript.

## DATA ACCESSIBILITY

DNA sequences (MHC class I α1 alleles) are available at GenBank (accession numbers MH706772 ‐ MH706834). Parasite‐mediated selection data (model set 1), MHC‐associated mating data (model set 2 and Fisher's exact test), signals of MHC genotype data (model set 3 and Fisher's exact tests), and relatedness and heterozygosity data have been deposited in the Dryad digital repository (https://doi.org/10.5061/dryad.mr31757).
