# Supplementary figures and images for: Specific MHC class I supertype associated with parasite infection and color morph in a wild lizard population
Source: Ecol Evol. 2018 Sep 17;8(19):9920–33. doi: 10.1002/ece3.4479 (PMC6202711; doi:10.1002/ece3.4479)

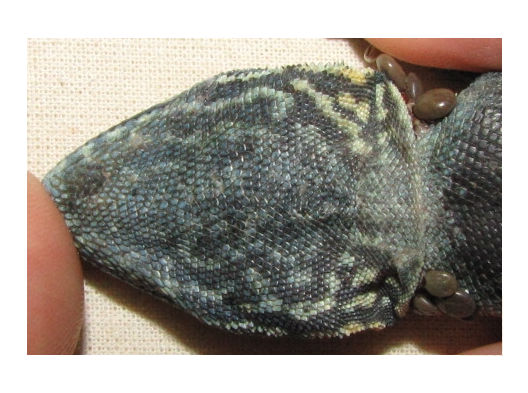

Supplement: Supplementary file 1 [file ECE3-8-9920-s001.tif]

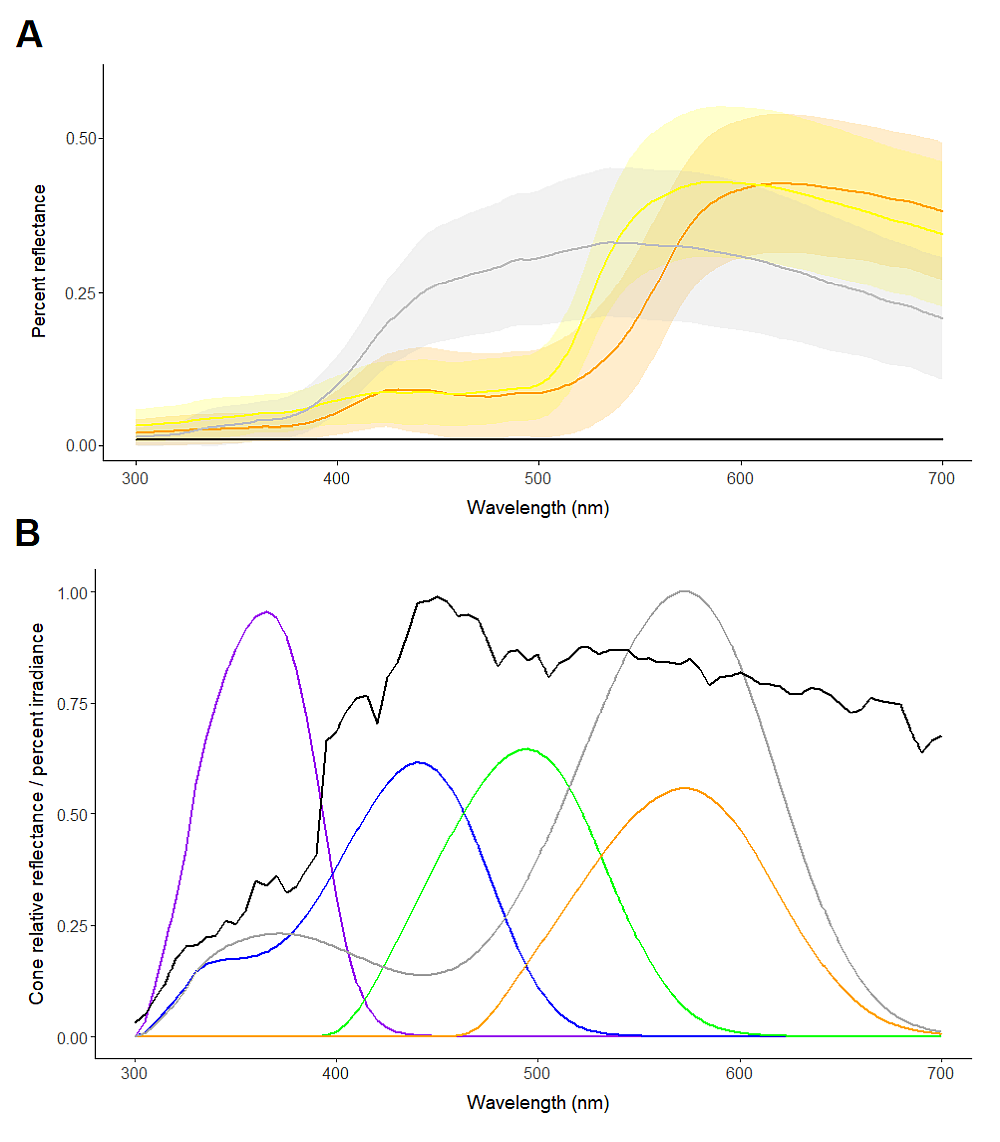

Supplement: Supplementary file 2 [file ECE3-8-9920-s002.tif]

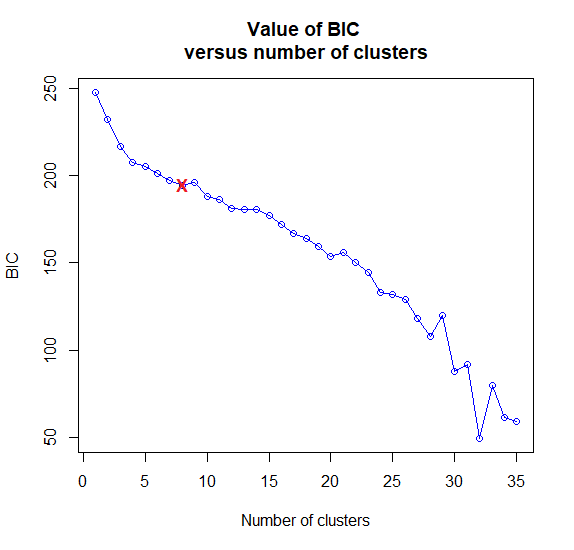

Supplement: Supplementary file 3 [file ECE3-8-9920-s003.tif]

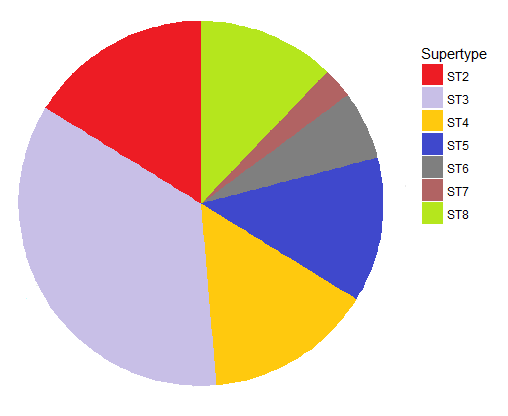

Supplement: Supplementary file 5 [file ECE3-8-9920-s005.tif]

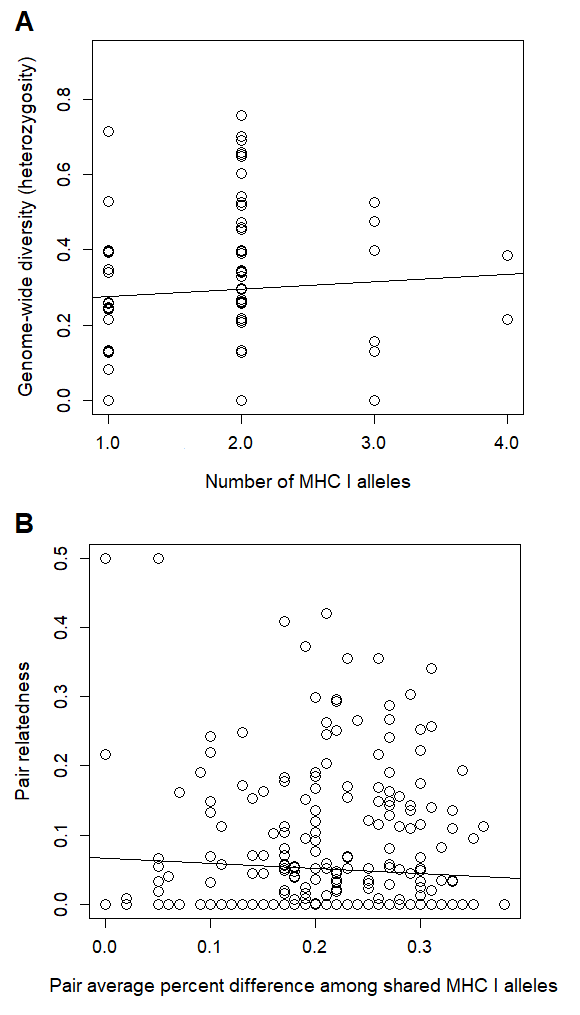

Supplement: Supplementary file 6 [file ECE3-8-9920-s006.tif]

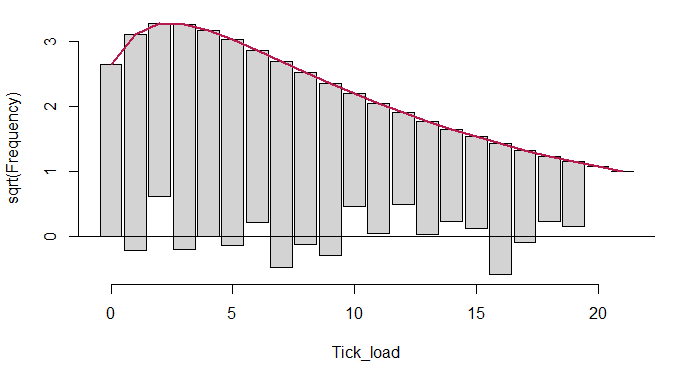

Supplement: Supplementary file 7 [file ECE3-8-9920-s007.tif]

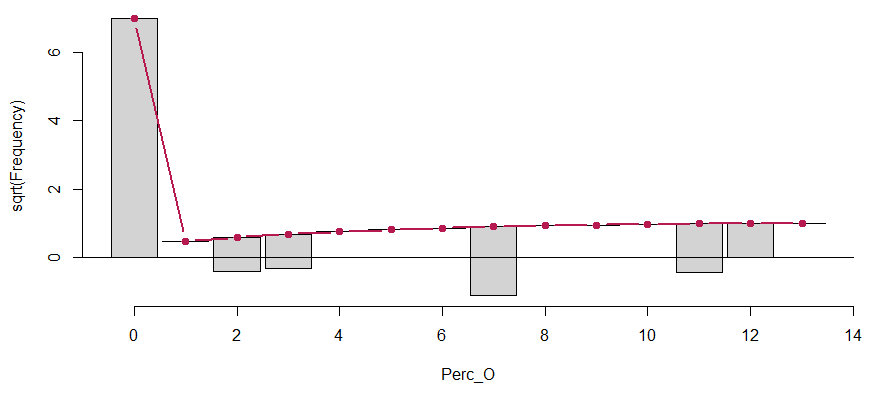

Supplement: Supplementary file 8 [file ECE3-8-9920-s008.tif]

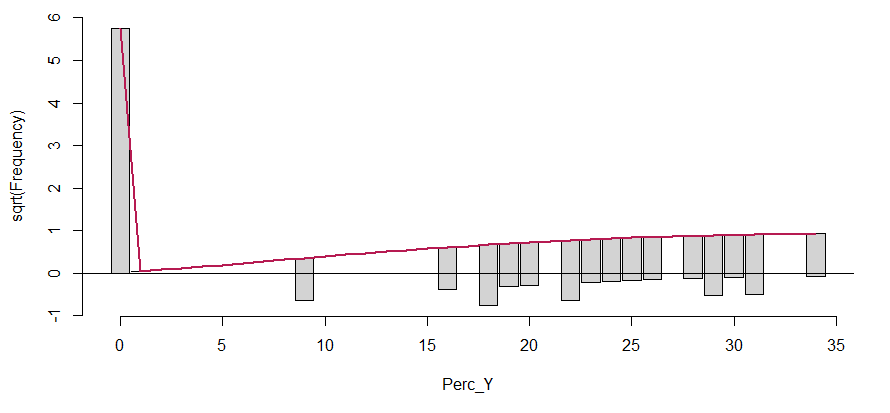

Supplement: Supplementary file 9 [file ECE3-8-9920-s009.tif]
