# Supplementary material for: Specific MHC class I supertype associated with parasite infection and color morph in a wild lizard population
Source: Ecol Evol. 2018 Sep 17;8(19):9920–33. doi: 10.1002/ece3.4479 (PMC6202711; doi:10.1002/ece3.4479)
Supplement: Supplementary file 14 [file ECE3-8-9920-s014.docx]

**Appendix**

**Specific MHC class I supertype associated with parasite infection and colour morph in a wild lizard population**

Jessica D. Hacking, Devi Stuart-Fox, Stephanie S. Godfrey, Michael G. Gardner

**Data S1.** Additional methodology for male throat colouration analysis

Measures of colouration are based on the visual system of agamid lizards (i.e. perception by conspecifics) and are independent of the human visual system. The proportion of yellow and orange throat colouration was derived from calibrated photographs by extracting portions of the photograph based on RGB values of each pixel according to set threshold values (Teasdale *et al.* 2013). For each male, photographs were taken at a standard distance and angle with a Canon© PowerShot SX50 HScamera. An X-Rite© ColourChecker Classic Cardcolour card was included in each photograph. Photographs were taken when males were at an active temperature (32°C ± 3°C). Each photograph was calibrated to adjust for differences in illumination among photographs using the RGBGys and reflectance values of the grey scale on the colour card as described in Teasdale *et al.* (2013). After calibration, photographs were cropped to include only the throat and the proportion of orange and yellow were calculated using threshold values of 0.35 and 0.20 for orange and yellow, respectively. Analysis was undertaken in Matlab (The MathWorks, Inc., Natick, MA, USA) using a custom program (Teasdale *et al.* 2013).

A portable Ocean Optics (Dunedin, FL, USA) Jaz spectrometer was used to collect two or three spectral reflectance measurements for each primary and secondary throat colour, holding the probe at a 45° angle to the throat. Measurements were taken every 1 nm within the visible spectrum of agamid lizards (300 to 700 nm) and were expressed relative to a white Spectralon (Labsphere, Inc., North Sutton, NH, USA) 99% diffuse reflectance standard. Males were at an active temperature (33°C ± 3°C) when measurements were taken. Reflectance measures (300 – 700 nm) were smoothed over 5 nm intervals using the Nadaraya–Watson kernel regression estimate implemented in R ver. 3.4.1 (R Core Team 2016) prior to analysis (fig. S2). After the relative stimulation of *C. decresii* photoreceptors (receptor quantum catches) were estimated (Vorobyev *et al.* 1998), we calculated the chromatic (colour) and achromatic (brightness) contrasts relative to a standard background (reflectance of 1%) under full sun irradiance (fig. S2). Chromatic and achromatic contrast was measured in units of ‘just noticeable differences’, with a just noticeable difference greater than three generally indicating a noticeable difference (here, from the standard background) from the perspective of the receiver (Vorobyev & Osorio 1998). Single cone sensitivities were used to calculate chromatic contrast and double cone sensitivity was used to calculate achromatic contrast (Yewers *et al.* 2015). Refer to (Teasdale *et al.* 2013) for further details on the visual models used here. Only the results for achromatic JNDs (‘brightness’) were used in subsequent statistical analyses. All calculations were undertaken in R, using the *pavo* package (Maia *et al.* 2013; R Core Team 2016).

To quantify chest patch size, photographs (as described above) were set to scale using the ruler included in each photograph and then the area of black/grey chest patch marking was calculated, using ImageJ ver. 1.46r (Schindelin *et al.* 2012). Relative chest patch size was calculated using residuals from a correlation of chest patch size and SVL.

**Data S2.** Results for model set 2 and Fisher’s exact test testing for MHC-associated mating

Three models were within the 95% confidence set for model set 2 (table S4). The model with the lowest ΔAICc included pair MHC I genetic distance only, the second model was the null model and the third was the full model, including both pair MHC I genetic distance and number of male MHC I alleles. The 95% CIs overlapped zero and effect sizes were small for both pair MHC I genetic distance (95% CIs -0.85, 2.35 and odds ratio 3.12, table S5, fig. S11) and number of male MHC I alleles (95% CIs -0.80, 0.68. and odds ratio 0.72, table S5, fig. S11). All top models had ΔAICc values <2 and evidence ratios were small (1.4 – 1.9), indicating that all models explained the data equally well. In regards to mate choice for specific MHC I supertypes, the Fisher’s exact test was non-significant (p-value = 0.11), indicating that the probability of possessing a certain supertype is independent of male mating status.

**Data S3.** Model set 3a – 3f results (male phenotypic signals of MHC genotype)

The 95% confidence set for model set 3a, in which number of MHC I alleles was the response variable, included nine models (tables S10 and S11). The top model (lowest ΔAICc) was the null model. All models had low ΔAICc and evidence ratios. Adjusted R^2^ values were very low for all models, indicating that the variables had little predictive power. Model set 3b, in which number of MHC I supertypes was the response variable, contained nine top models (95% confidence set, tables S12 and S13). All models within the 95% confidence set included percentage of male throat coloured yellow (PercY) and the top model included only PercY. PercY was highest in individuals with one supertype and lowest in individuals with three supertypes. All top models had low ΔAICc and ERs, hence there was no clear ‘best’ model. All models were poor fits, with adjusted R^2^ reaching a maximum of 0.18 among the top model set.

Model sets 3c and 3d uncovered no effect of the presence of certain supertypes on percentage of male throat coloured orange (PercO) and percentage of male throat coloured yellow (PercY). Rootograms showed that hurdle models fit well in both cases, although they suffered from slight underestimation (figs. S8 and S9). The 95% confidence set for model set 3c (PercO as response) included four models that had a ΔAICc less than two (tables S6 and S7, fig. S12). These models included supertypes four and six, with PercO lower in the presence of both supertypes. This association was driven by the zero (binomial) component of the hurdle model, revealing that the presence of supertypes four and six was associated with the absence of orange colour on the throat rather than the amount of orange colour on the throat. Although supertypes four and six were present in the top models and the hurdle model fitted the data well, 95% CIs included zero for both supertypes and effect sizes were small. In regard to model set 3d (PercY as response), the 95% confidence set included five models for which ΔAICc was less than two (tables S8 and S9, fig. S13). These models included supertypes three, four and five. Again, these associations were driven by the zero (binomial) component of the hurdle model, indicating that the presence or absence of yellow on the throat, rather than the amount of yellow on the throat, is associated with certain supertypes. Supertypes three, four and five all displayed a negative relationship with PercY. Supertype five had the largest effect size, although overall effect sizes were small, and 95% CIs overlapped zero for all supertypes.

Model sets 3e (throat brightness as response) and 3f (chest patch as response) revealed little evidence that throat brightness and chest patch are associated with certain MHC I supertypes. In model set 3e the null model had the lowest ΔAICc and model fit was poor (tables S14 and S15). Model fit was also poor for model set 3f, although supertypes four and six had lower ΔAICc than the null model (tables S16 and S17). The 95% CIs overlapped zero for all variables in each model set.

**Table S1.** Summary of statistical tests used to test hypotheses regarding the mechanisms shaping MHC class I diversity within a wild *C. decresii* population. See table S2 for variable descriptions.

| Model set | Response | Predictors | OPV* |
| --- | --- | --- | --- |
| *Parasite-mediated selection* | | | |
| 1, hurdle model | Tick load | Supertypes: ST2 – ST8; *covariates:* year, time in season | 15 |
| *MHC-associated mating* | | | |
| 2, GLMM | Mating status | Pair MHC I genetic distance, number MHC alleles (male); *covariates:* pair spatial proximity, male mass; *random factors:* male ID, female ID | 4 |
|  |  |  |  |
| Fisher’s exact test | Mating status | Prevalence of supertypes ST2 – ST8 | |
| *Signals of MHC genotype* | | | |
| 3a, GLM | Number MHC alleles | Percentage of throat coloured orange, percentage of throat coloured yellow, throat brightness, relative chest patch size | 14 |
| 3b, GLM | Number MHC supertypes |  |  |
| 3c, hurdle model | Percentage of throat coloured orange | Supertypes ST2 – ST8 | 12 |
| 3d, hurdle model | Percentage of throat coloured yellow |  | 12  10 |
| 3e, GLM | Throat brightness |  |  |
| 3f, GLM | Relative chest patch size |  | 12 |
| 3g, GLM | Mating status | Percentage of throat coloured orange, percentage of throat coloured yellow; *co-variate:* male mass | 5 |
| Fisher’s exact test | Supertype ST4 | Morph types |  |
| Fisher’s exact test | Mating status | Morph types |  |

*OPV: Observations per variable: The number of observations divided by the number of predictor variables (including co-variates). For binary response variables the number of observations is equal to the smaller of i) the number of events (i.e. mated) or ii) the number non-events (i.e. available).

**Table S2.** Description of variables used in hypothesis testing. Ordered according to table S1.

| Variable | Abbreviation | Description |
| --- | --- | --- |
| Tick load | TL | The number of ticks attached to an individual |
| Supertypes |  | Each supertype (ST2 to ST8) is a separate variable, binary coded for absence or presence |
| Year |  | The year, or season, in which sampling took place. |
| Time in season | Season | The time during the season (early or late) that sampling took place. |
| Mating status |  | Each male either mated with, or was available to (within 100m radius), a given female |
| Pair MHC I genetic distance | Al_dist | The average percent genetic (amino acid) distance between shared MHC I alleles of a male and female |
| Number MHC alleles (male) | Male_Al | The number of MHC I alleles per individual male. |
| Number MHC supertypes (male) | Male_ST | The number of MHC I supertypes per individual male. |
| Pair spatial proximity | Geo_dist | Shortest geographic distance (m) between a male and female |
| Male mass |  | Male mass (g) |
| Percentage of throat coloured orange | PercO | Percentage of male throat coloured orange |
| Percentage of throat coloured yellow | PercY | Percentage of male throat coloured yellow |
| Throat brightness | TB | Achromatic JNDs of male throat colour against a standard background, a measure of brightness |
| Relative chest patch size | CP | Male chest patch size relative to SVL |
| Morph types |  | Male throat morph type; yellow, yellow/orange, orange and grey |

**Table S3.** Summary results for variables included in model set 1 top (95% confidence set) models (response: tick load, predictors: MHC I supertypes), after model averaging. See main text for model results. Bolded and italicised CI values do not overlap zero. See table S2 for variable descriptions and abbreviations.

| Variable | Estimate | Adjusted SE | Lower CI | Upper CI | Relative importance |
| --- | --- | --- | --- | --- | --- |
| Count_ST4 | -0.42 | 0.31 | -0.98 | -0.15 | 0.74 |
| Zero_ST4 | -0.26 | 0.81 | -2.15 | 1.45 | 0.74 |
| Count_ST3 | 0.07 | 0.15 | -0.06 | 0.61 | 0.27 |
| Zero_ST3 | 0.15 | 0.50 | -1.10 | 2.19 | 0.27 |
| Zero_ST7 | 2.07 | 1.08+03 | -5.67+03 | 5.70+03 | 0.14 |
| Count_ST7 | -0.06 | 0.21 | -1.24 | 0.39 | 0.14 |
| Zero_ST2 | -0.03 | 0.38 | -2.04 | 1.68 | 0.16 |
| Count_ST2 | -0.04 | 0.12 | -0.63 | 0.11 | 0.16 |
| Zero_ST5 | -0.07 | 0.41 | -2.26 | 1.38 | 0.17 |
| Count_ST5 | -0.04 | 0.13 | -0.66 | 0.12 | 0.17 |
| Zero_ST6 | -0.03 | 0.40 | -2.48 | 2.00 | 0.12 |
| Count_ST6 | -0.03 | 0.13 | -0.80 | 0.21 | 0.12 |
| Zero_ST8 | -0.28 | 0.67 | -3.07 | 0.39 | 0.21 |
| Count_ST8 | 0.00 | 0.10 | -0.43 | 0.44 | 0.21 |
| Count_Year (covariate) | -0.39 | 0.22 | -0.82 | 0.03 | 1.00 |
| Zero_Year (covariate) | -0.28 | 0.91 | -2.06 | 1.50 | 1.00 |
| Zero_Time in season (covariate) | 0.15 | 1.18 | -2.16 | 2.46 | 1.00 |
| Count_Time in season (covariate) | 0.40 | 0.21 | -0.02 | 0.82 | 1.00 |

**Table S4.** AIC information-theoretic top (95% confidence set) model selection results for model set 2 (response: male mating status, predictors: MHC I diversity and MHC I genetic distance), after model averaging. The null model includes only covariables (male mass and pair spatial proximity). See table S2 for variable descriptions and abbreviations. ER = evidence ratio.

| Model | df | AICc | ΔAICc | Weight | ER | R^2^_m_ | R^2^_c_ |
| --- | --- | --- | --- | --- | --- | --- | --- |
| Al_dist | 6 | 110.08 | 0.00 | 0.48 |  | 0.002 | 0.025 |
| Null | 5 | 110.74 | 0.66 | 0.34 | 1.4 | 0.002 | 0.021 |
| Al_dist + Male_Al | 7 | 111.99 | 1.91 | 0.18 | 1.9 | 0.488 | 0.599 |

**Table S5.** Summary results for variables included in model set 2 top (95% confidence set) models (response: male mating status, predictors: MHC I diversity and MHC I genetic distance), after model averaging. Effect sizes standardised on two S.D. See main text for model results. See table S2 for variable descriptions and abbreviations.

| **Variable** | **Estimate** | **Adjusted SE** | **Lower CI** | **Upper CI** | **Relative importance** |
| --- | --- | --- | --- | --- | --- |
| Al_dist | 0.75 | 0.81 | -0.85 | 2.35 | 0.66 |
| Male_Al | -0.06 | 0.38 | -0.80 | 0.68 | 0.18 |
| Male_mass (covariate) | 2.32 | 0.84 | 0.66 | 3.97 | 1.00 |
| Geo_dist (covariate) | -3.04 | 0.77 | -4.54 | -1.54 | 1.00 |

**Table S6.** AIC information-theoretic top model selection results for model set 3c (response: percentage of male throat coloured orange, predictors: MHC I supertypes), after model averaging. Only those models with ΔAICc ≤2 are shown due to the large number of models in the 95% confidence set. See table S2 for variable descriptions and abbreviations. ER = evidence ratio.

| Model | df | AICc | ΔAICc | Weight | ER |
| --- | --- | --- | --- | --- | --- |
| ST4 + ST6 | 7 | 447.02 | 0.00 | 0.19 |  |
| ST6 | 5 | 447.19 | 0.17 | 0.17 | 1.1 |
| Null | 3 | 447.84 | 0.82 | 0.12 | 1.4 |
| ST4 | 5 | 448.58 | 1.57 | 0.09 | 1.3 |

**Table S7.** Summary results for predictor variables included in model set 3c top (95% confidence set) models (response: male percentage throat coloured orange, predictors: MHC I supertypes), after model averaging. See main text for model results. See table S2 for variable descriptions and abbreviations.

| Variable | Estimate | Adjusted SE | Lower CI | Upper CI | Relative importance |
| --- | --- | --- | --- | --- | --- |
| Count_ST4 | -0.13 | 0.25 | -0.83 | 0.48 | 0.46 |
| Zero_ST4 | -0.45 | 0.61 | -1.73 | 0.72 | 0.46 |
| Count_ST6 | -0.15 | 0.58 | -1.80 | 0.93 | 0.61 |
| Zero_ST6 | -1.28 | 1.33 | -3.08 | 1.08 | 0.61 |
| Count_ST5 | 0.03 | 0.12 | -0.40 | 0.57 | 0.12 |
| Zero_ST5 | 0.02 | 0.20 | -0.62 | 0.49 | 0.12 |
| Count_ST2 | -0.01 | 0.09 | -0.24 | 0.23 | 0.08 |
| Zero_ST2 | -0.01 | 0.16 | -0.45 | 0.38 | 0.08 |
| Count_ST3 | -0.01 | 0.08 | -0.18 | 0.19 | 0.08 |
| Zero_ST3 | 0.01 | 0.14 | -0.31 | 0.27 | 0.08 |
| Count_ST7 | 0.02 | 0.15 | -0.45 | 0.52 | 0.07 |
| Zero_ST7 | 0.02 | 0.30 | -0.60 | 0.58 | 0.07 |
| Count_ST8 | 0.01 | 0.09 | -0.20 | 0.23 | 0.09 |
| Zero_ST8 | 0.01 | 0.17 | -0.30 | 0.33 | 0.09 |

**Table S8.** AIC information-theoretic top model selection results for model set 3d (response: percentage of male throat coloured yellow, predictors: MHC I supertypes), after model averaging. Only those models with ΔAICc ≤2 are shown due to the large number of models in the 95% confidence set. See table S2 for variable descriptions and abbreviations. ER = evidence ratio.

| Model | df | AICc | ΔAICc | Weight | ER |
| --- | --- | --- | --- | --- | --- |
| ST3 + ST5 | 7 | 624.56 | 0.00 | 0.12 |  |
| ST3 | 5 | 624.60 | 0.04 | 0.12 | 1.0 |
| Null | 3 | 624.92 | 0.36 | 0.10 | 1.2 |
| ST3 + ST5 + ST4 | 9 | 625.42 | 0.86 | 0.08 | 1.3 |
| ST5 | 5 | 626.10 | 1.55 | 0.06 | 1.3 |

**Table S9.** Summary results for predictor variables included in model set 3d top (95% confidence set) models (response: male percentage throat coloured yellow, predictors: MHC I supertypes), after model averaging. See main text for model results. See table S2 for variable descriptions and abbreviations.

| Variable | Estimate | Adjusted SE | Lower CI | Upper CI | Relative importance |
| --- | --- | --- | --- | --- | --- |
| Count_ST3 | -0.17 | 0.18 | -0.51 | 0.18 | 0.60 |
| Zero_ST3 | -0.36 | 0.51 | -1.35 | 0.63 | 0.60 |
| Count_ST5 | -0.06 | 0.15 | -0.36 | 0.24 | 0.48 |
| Zero_ST5 | -0.59 | 0.76 | -2.08 | 0.90 | 0.48 |
| Count_ST4 | 0.00 | 0.09 | -0.17 | 0.17 | 0.26 |
| Zero_ST4 | -0.23 | 0.48 | -1.17 | 0.70 | 0.26 |
| Count_ST2 | -0.02 | 0.07 | -0.16 | 0.13 | 0.15 |
| Zero_ST2 | 0.08 | 0.34 | -0.57 | 0.74 | 0.15 |
| Count_ST8 | 0.01 | 0.07 | -0.13 | 0.14 | 0.12 |
| Zero_ST8 | 0.07 | 0.29 | -0.50 | 0.64 | 0.12 |
| Count_ST6 | -0.01 | 0.07 | -0.15 | 0.14 | 0.10 |
| Zero_ST6 | 0.07 | 0.35 | -0.61 | 0.75 | 0.10 |
| Count_ST7 | 0.00 | 0.10 | -0.20 | 0.19 | 0.07 |
| Zero_ST7 | -0.04 | 0.31 | -0.64 | 0.56 | 0.07 |

**Table S10.** AIC information-theoretic top (95% confidence set) model selection results for model set 3a (response: number MHC I alleles, predictors: potential signals of MHC diversity), after model averaging. See table S2 for variable descriptions and abbreviations. ER = evidence ratio.

| Model | df | AICc | ΔAICc | Weight | ER | Adj-R^2^ |
| --- | --- | --- | --- | --- | --- | --- |
| Null | 2 | 131.94 | 0.00 | 0.23 |  | 0.00 |
| PercY | 3 | 132.69 | 0.76 | 0.16 | 1.5 | 0.03 |
| CP | 3 | 133.63 | 1.70 | 0.10 | 1.6 | 0.01 |
| TB | 3 | 134.02 | 2.08 | 0.08 | 1.2 | 0.00 |
| PercO | 3 | 134.15 | 2.21 | 0.08 | 1.1 | 0.00 |
| PercY + CP | 4 | 134.58 | 2.65 | 0.06 | 1.2 | 0.04 |
| PercY + TB | 4 | 134.65 | 2.71 | 0.06 | 1.0 | 0.04 |
| PercY + PercO | 4 | 134.77 | 2.83 | 0.06 | 1.1 | 0.03 |
| CP + TB | 4 | 135.73 | 3.80 | 0.03 | 1.6 | 0.01 |

**Table S11.** Summary results for predictor variables included in model set 3a top (95% confidence set) models (response: number MHC I alleles, predictors: potential signals of MHC diversity), after model averaging. Effect sizes standardised on two S.D. See table S2 for variable descriptions and abbreviations.

| Variable | Estimate | Adjusted SE | Lower CI | Upper CI | Relative importance |
| --- | --- | --- | --- | --- | --- |
| PercY | -0.10 | 0.18 | -0.44 | 0.25 | 0.39 |
| CP | 0.03 | 0.11 | -0.19 | 0.26 | 0.23 |
| TB | 0.02 | 0.10 | -0.18 | 0.22 | 0.20 |
| PercO | -0.01 | 0.09 | -0.18 | 0.16 | 0.15 |

**Table S12.** AIC information-theoretic top (95% confidence set) model selection results for model set 3b (response: number of MHC I supertypes, predictors: potential signals of MHC diversity), after model averaging. See table S2 for variable descriptions and abbreviations. ER = evidence ratio.

| Model | df | AICc | ΔAICc | Weight | ER | Adj-R^2^ |
| --- | --- | --- | --- | --- | --- | --- |
| PercY | 3 | 121.25 | 0.00 | 0.24 |  | 0.12 |
| PercY + TB | 4 | 121.64 | 0.39 | 0.19 | 1.2 | 0.15 |
| PercY + CP | 4 | 122.63 | 1.38 | 0.12 | 1.6 | 0.14 |
| PercY + TB + CP | 5 | 122.81 | 1.56 | 0.11 | 1.1 | 0.17 |
| PercY + PercO | 4 | 122.98 | 1.73 | 0.10 | 1.1 | 0.13 |
| PercY + TB + PercO | 5 | 123.79 | 2.54 | 0.07 | 1.5 | 0.16 |
| PercY + CP + PercO | 5 | 124.57 | 3.33 | 0.04 | 1.5 | 0.14 |
| PercY + TB + CP + PercO | 6 | 125.17 | 3.92 | 0.03 | 1.3 | 0.18 |
| Null | 2 | 125.32 | 4.07 | 0.03 | 1.1 | 0.00 |

**Table S13.** Summary results for predictor variables included in model set 3b top (95% confidence set) models (response: number of MHC I supertypes, predictors: potential signals of MHC diversity), after model averaging. Effect sizes standardised on two S.D. See table S2 for variable descriptions and abbreviations.

| Variable | Estimate | Adjusted SE | Lower CI | Upper CI | Relative importance |
| --- | --- | --- | --- | --- | --- |
| PercY | -0.46 | 0.20 | -0.86 | -0.06 | 0.97 |
| TB | 0.11 | 0.17 | -0.23 | 0.45 | 0.43 |
| CP | 0.06 | 0.14 | -0.21 | 0.32 | 0.33 |
| PercO | -0.03 | 0.11 | -0.25 | 0.19 | 0.26 |

**Table S14.** AIC information-theoretic top (95% confidence set) model selection results for model set 3e (response: throat brightness, predictors: supertypes), after model averaging. Only models with a ΔAICc ≤2 are shown due to the high number of models in the 95% confidence set. See table S2 for variable descriptions and abbreviations. ER = evidence ratio.

| Model | df | AICc | ΔAICc | Weight | ER | Adj-R^2^ |
| --- | --- | --- | --- | --- | --- | --- |
| Null | 2 | 468.64 | 0.00 | 0.07 |  | 0.00 |
| ST8 | 3 | 469.63 | 0.99 | 0.04 | 1.8 | 0.02 |
| ST2 | 3 | 469.90 | 1.27 | 0.04 | 1.0 | 0.01 |
| ST3 | 3 | 469.92 | 1.28 | 0.04 | 1.0 | 0.01 |
| ST7 | 3 | 470.27 | 1.63 | 0.03 | 1.3 | 0.01 |
| ST5 | 3 | 470.40 | 1.76 | 0.03 | 1.0 | 0.01 |
| ST2 + ST5 | 4 | 470.55 | 1.92 | 0.03 | 1.0 | 0.04 |
| ST4 | 3 | 470.60 | 1.96 | 0.03 | 1.0 | 0.00 |

**Table S15.** Summary results for predictor variables included in model set 3e top (95% confidence set) models (response: throat brightness, predictors: supertypes), after model averaging. Effect sizes standardised on two S.D. See table S2 for variable descriptions and abbreviations.

| Variable | Estimate | Adjusted SE | Lower CI | Upper CI | Relative importance |
| --- | --- | --- | --- | --- | --- |
| ST8 | 0.92 | 1.89 | -2.78 | 4.62 | 0.36 |
| ST2 | 1.13 | 1.85 | -2.49 | 4.75 | 0.44 |
| ST3 | -0.43 | 1.16 | -2.70 | 1.85 | 0.30 |
| ST7 | 0.92 | 2.36 | -3.72 | 5.55 | 0.30 |
| ST5 | -0.76 | 1.72 | -4.14 | 2.61 | 0.34 |
| ST4 | 0.34 | 1.13 | -1.87 | 2.55 | 0.27 |
| ST6 | 0.15 | 1.42 | -2.62 | 2.93 | 0.22 |

**Table S16.** AIC information-theoretic top (95% confidence set) model selection results for model set 3f (response: chest patch size, predictors: supertypes), after model averaging. Only models with a ΔAICc ≤2 are shown due to the high number of models in the 95% confidence set. See table S2 for variable descriptions and abbreviations. ER = evidence ratio.

| Model | df | AICc | ΔAICc | Weight | ER | Adj-R^2^ |
| --- | --- | --- | --- | --- | --- | --- |
| ST4 | 3 | 197.91 | 0.00 | 0.07 |  | 0.04 |
| ST4 + ST6 | 4 | 198.79 | 0.87 | 0.04 | 1.8 | 0.06 |
| Null | 2 | 198.88 | 0.97 | 0.04 | 1.0 | 0.00 |
| ST4 + ST8 | 4 | 199.25 | 1.34 | 0.03 | 1.3 | 0.05 |
| ST4 + ST3 | 4 | 199.41 | 1.50 | 0.03 | 1.0 | 0.05 |
| ST4 + ST2 | 4 | 199.46 | 1.55 | 0.03 | 1.0 | 0.05 |
| ST2 | 3 | 199.50 | 1.58 | 0.03 | 1.0 | 0.02 |

**Table S17.** Summary results for predictor variables included in model set 3f top (95% confidence set) models (response: chest patch size, predictors: supertypes), after model averaging. Effect sizes standardised on two S.D. See table S2 for variable descriptions and abbreviations.

| Variable | Estimate | Adjusted SE | Lower CI | Upper CI | Relative importance |
| --- | --- | --- | --- | --- | --- |
| ST4 | 0.20 | 0.23 | -0.26 | 0.66 | 0.58 |
| ST6 | 0.10 | 0.24 | -0.36 | 0.57 | 0.33 |
| ST8 | 0.04 | 0.14 | -0.24 | 0.32 | 0.27 |
| ST3 | -0.05 | 0.13 | -0.31 | 0.21 | 0.31 |
| ST2 | -0.07 | 0.16 | -0.38 | 0.24 | 0.33 |
| ST5 | 0.00 | 0.12 | -0.24 | 0.24 | 0.23 |
| ST7 | 0.01 | 0.20 | -0.38 | 0.39 | 0.22 |

**Table S18.** AIC information-theoretic top (95% confidence set) model selection results for model set 3g (response: mating status, predictors: male percentage throat coloured orange and male percentage throat coloured yellow), after model averaging. The null model includes only co-variates (male mass). See table S2 for variable descriptions and abbreviations. ER = evidence ratio.

| Model | df | AICc | ΔAICc | Weight | ER | Adj-R^2^ |
| --- | --- | --- | --- | --- | --- | --- |
| Null | 2 | 88.85 | 0.00 | 0.55 |  | 0.12 |
| PercY | 3 | 90.37 | 1.52 | 0.26 | 2.1 | 0.13 |
| PercO | 3 | 90.96 | 2.11 | 0.19 | 1.4 | 0.12 |

**Table S19.** Summary results for predictor variables included in model set 3g top (95% confidence set) models (response: mating status, predictors: male percentage throat coloured orange and male percentage throat coloured yellow), after model averaging. Effect sizes standardised on two S.D. See table S2 for variable descriptions and abbreviations.

| Variable | Estimate | Adjusted SE | Lower CI | Upper CI | Relative importance |
| --- | --- | --- | --- | --- | --- |
| PercY | -0.11 | 0.36 | -0.82 | 0.59 | 0.26 |
| PercO | 0.01 | 0.26 | -0.50 | 0.51 | 0.19 |
| Male mass (co-variable) | 1.66 | 0.65 | 0.40 | 2.93 | 1.00 |

**References**

Jombart T, Devillard S, Balloux F (2010) Discriminant analysis of principal components: a new method for the analysis of genetically structured populations. *BMC Genetics* **11**, 94.

Maia R, Eliason CM, Bitton P-P, Doucet SM, Shawkey MD (2013) pavo: an R package for the analysis, visualization and organization of spectral data. *Methods in Ecology and Evolution* **4**, 906-913.

R Core Team (2016) R: A language and environment for statistical computing. R Foundation for Statistical Computing, Vienna, Austria.

Schindelin J, Arganda-Carreras I, Frise E*, et al.* (2012) Fiji: an open-source platform for biological-image analysis. *Nature Methods* **9**, 676-682.

Teasdale L, Stevens M, Stuart-Fox D (2013) Discrete colour polymorphism in the tawny dragon lizard (*Ctenophorus decresii*) and differences in signal conspicuousness among morphs. *Journal of Evolutionary Biology* **26**.

Vorobyev M, Osorio D (1998) Receptor noise as a determinant of colour thresholds. *Proceedings of the Royal Society B: Biological Sciences* **265**, 351-358.

Vorobyev M, Osorio D, Bennett ATD, Marshall NJ, Cuthill IC (1998) Tetrachromacy, oil droplets and bird plumage colours. *Journal of Comparative Physiology A* **183**, 621-633.

Yewers MS, McLean CA, Moussalli A*, et al.* (2015) Spectral sensitivity of cone photoreceptors and opsin expression in two colour-divergent lineages of the lizard *Ctenophorus decresii*. *The Journal of Experimental Biology* **218**, 1556-1563.
